# Comparison of safety and effectiveness between robotic and laparoscopic major hepatectomy: a systematic review and meta-analysis

**DOI:** 10.1097/JS9.0000000000000750

**Published:** 2023-09-14

**Authors:** Benliang Mao, Shanfei Zhu, Dan Li, Junhao Xiao, Bailin Wang, Yong Yan

**Affiliations:** Departments of a General Surgery; b Thoracic Surgery, Guangzhou Red Cross Hospital, Jinan University, Guangzhou; c College of Clinical Medicine, Guizhou Medical University, Guiyang, China

**Keywords:** laparoscopic hepatectomy, major hepatectomy, meta-analysis, robotic hepatectomy

## Abstract

**Background:**

Robotic platform has been increasingly applied in major hepatectomy. However, the role or advantage of robotic approach comparing with laparoscopic approach in major hepatectomy remains controversial. This meta-analysis compares perioperative outcomes of robotic major hepatectomy (RMH) to laparoscopic major hepatectomy (LMH) for hepatic neoplasms.

**Methods:**

PubMed, Web of Science, EMBASE, and Cochrane Library were searched to identify comparative studies compared RMH versus LMH for hepatic neoplasms. The search timeframe was set before May 2023. Main outcomes were mortality, overall morbidities, serious complications, and conversion to open surgery. Secondary outcomes were operative time, intraoperative blood loss, blood transfusion, postoperative length of hospital stay, R0 resection, reoperation, and readmission. Studies were evaluated for quality by Cochrane risk of bias tool or Newcastle-Ottawa scale. Data were pooled as odds ratio (OR) or mean difference (MD). This study was registered at PROSPERO (CRD42023410951).

**Results:**

Twelve retrospective cohort studies concerning total 1657 patients (796 RMH, 861 LMH) were included. Meta-analyses showed no significant differences in mortality (OR=1.23, 95% CI=0.50–2.98, *P*=0.65), overall postoperative complications (OR=0.83, 95% CI=0.65–1.06, *P*=0.14), operative time (MD=6.47, 95% CI=−14.72 to 27.65, *P*=0.55), blood transfusion (OR=0.77, 95% CI=0.55–1.08, *P*=0.13), R0 resection (OR=1.45, 95% CI=0.91–2.31, *P*=0.12), reoperation (OR=0.76, 95% CI=0.31–1.88, *P*=0.56), and readmission (OR=0.63, 95% CI=0.28–1.44, *P*=0.27) between RMH and LMH. Incidence of serious complications (OR=0.60, 95% CI=0.40–0.90, *P*=0.01), conversion to open surgery (OR=0.41, 95% CI=0.27–0.63, *P*<0.0001), blood loss (MD=−91.42, 95% CI=−142.18 to −40.66, *P*=0.0004), and postoperative hospital stay (MD=−0.64, 95% CI=−0.78 to −0.49, *P*<0.00001) were reduced for RMH versus LMH.

**Conclusions:**

RMH is associated with comparable short-term surgical outcomes and oncologic adequacy compared to LMH when performed by experienced surgeons at large centres. RMH may result in reduced major morbidities, conversion rate, blood loss, and hospital stay, but these results were volatile. Further randomized studies should address the potential advantages of RMH over LMH.

## Introduction

Highlights

- A meta-analysis to compare robotic and laparoscopic major hepatectomy, and twelve published studies were included.
- Robotic major hepatectomy (RMH) is associated with comparable short-term surgical outcomes and oncologic adequacy compared to laparoscopic major hepatectomy (LMH).
- RMH may result in reduced serious complications, conversion rate, blood loss, and hospital stay compared to LMH.
- Further randomized trials should address the potential advantages of RMH over LMH.

Liver resection is one of the most common surgical treatments for hepatic neoplasms, which can be divided into two types, namely minimally invasive liver resection and conventional open liver resection^1^. Generally, minimally invasive liver resection included traditional laparoscopic and newer robotic approaches. Over the past decade, minimally invasive liver resection has trended from laparoscopic toward robotic platform^2^. Owing to the technical benefits of 7 degrees of freedom, improved visibility, and enhanced ergonomics, robotic liver resection has gained popularity globally and it is gradually becoming a competent alternative procedure, especially for complex hepatectomy in high volume centres with proficient surgeons^3^. Despite improved technique and instruments, minimally invasive liver resection remains one of the most challenging procedures of hepatic surgery, as well as both laparoscopic and robotic hepatectomies are currently performed by experienced surgical teams in referral centres^4^.

Despite minimally invasive liver resection has been widely adopted, the early experience with minimally invasive liver resection was mostly based on minor liver resections^5^, defined as the resection of less than three Couinaud segments according to the Brisbane 2000 nomenclature^6^. During the past decade, there has been an increasing effort to perform more complex major hepatectomy with minimally invasive approach. As the accumulation of surgical experience and development of surgical instruments, minimally invasive surgery has also been used in major hepatectomy, it has been proven to be safe and feasible compared with open approach^7–9^. However, minimally invasive procedure in such complex condition often arouses concerns including higher risk for uncontrolled haemorrhage, suboptimal surgical instruments for major hepatectomy, the requirement of advanced technical expertise, and prolonged operative time^10^.

The robotic platform was developed to overcome some of the disadvantages in laparoscopic surgery along with three-dimensional operative vision and improved dexterity. The three-dimensional view and flexible instruments are allowing the surgeon to perform delicate tissue dissection, it seems logical that these theoretical advantages may translate into improved surgical outcomes especially for more complex major hepatectomy^11^. In recent years, a series of cohort studies and subsequent meta-analyses have demonstrated the comparable mortality, morbidity, and oncologic adequacy of robotic hepatectomy compared with laparoscopic hepatectomy^12–17^. However, most reports dominated by minor hepatectomies or combined major-minor series, the effects of robotic major hepatectomy (RMH) versus laparoscopic major hepatectomy (LMH) on perioperative and oncologic outcomes are still controversial. Despite there were two meta-analyses assessed surgical outcomes between RMH and LMH in the past 3 years^18,19^, which included few small sample articles and had conflicting pooled results. This meta-analysis aims to assess the potential advantages of robotic platform compared with laparoscopic approach for major hepatectomy using all the available published studies, while focusing on perioperative outcomes such as mortality, morbidity, conversion to open surgery, operative time, blood loss, blood transfusion, hospital stay, R0 resection, reoperation, and readmission.

## Methods

This work has been reported in line with Preferred Reporting Items for Systematic Reviews and Meta-Analyses (PRISMA, Supplemental Digital Content 1, http://links.lww.com/JS9/A1000, Supplemental Digital Content 2, http://links.lww.com/JS9/B2) statement and Assessing the Methodological Quality of Systematic Reviews (AMSTAR) guidelines^20,21^, Supplemental Digital Content 3, http://links.lww.com/JS9/B3. This study was registered at PROSPERO (CRD42023410951).

### Search strategy

We searched the English-language literature published up to May 2023 in PubMed, Web of Science, EMBASE, and Cochrane Library. The search terms were: [laparoscopic OR laparoscopy OR laparoscopic-assisted OR minimally invasive OR minimal invasive surgery] AND [robot OR robotic OR robotic-assisted OR da vinci] AND [hepatectomy OR liver resection OR hepatic surgery OR liver surgery OR major hepatectomy OR hemihepatectomy OR posterosuperior sectionectomies OR right anterior sectionectomies OR right posterior sectionectomies] AND [randomized controlled trial OR prospective study OR comparative study OR retrospective study]. Two authors independently conducted the literature search and cross checked.

### Inclusion and exclusion criteria

According to Brisbane classification, major hepatectomy includes the following: (1) resection of 3 or more Couinaud segments or (2) resection of the right posterior, right anterior section because of the unique techniques required^22,23^. The articles evaluating robotic and laparoscopic procedures for major hepatectomy were considered eligible. Two authors screened eligible articles independently according the inclusion criteria: (1) the interventions compared were included RMH versus LMH; (2) elective hepatectomy for liver neoplasms; (3) adults patients; and (4) reported perioperative outcomes. The exclusion criteria were as follows: (1) laparoscopic hepatectomy performed by hand-assist method or hybrid approach; (2) living donor hepatectomy for liver transplantation; (3) experimental or animal studies; (4) single surgical technique with no comparative data; (5) studies without perioperative data; (6) emergency hepatectomy for abdominal trauma; and (7) the publication type was editorial, abstract, letter, case report, and expert opinion.

### Data extraction

The data were extracted using a predefined data extraction sheet. Both authors independently extracted data and then cross checked. In case of inconsistencies, a third author was consulted to reach a consensus. Parameters such as study characteristics, demographic characteristics, baseline characteristics, baseline matching status, methodological quality, included diseases, surgical details, surgical centres, and surgeons’ experience were extracted. The primary outcomes were postoperative mortality, overall postoperative complications, serious postoperative complications, and conversion to open surgery. Furthermore, secondary outcomes comprised operative time, interoperative blood loss, blood transfusion, postoperative length of hospital stay, R0 resection, reoperation, and unplanned readmission.

### Quality assessment

Two investigators independently assessed the risk of bias in the included studies and cross validated the results. The Newcastle-Ottawa scale (NOS) was used to assess the risk of bias for cohort studies with some modifications to match the needs of this study^24,25^, and the Cochrane risk bias tool for randomized controlled trial where applicable. The quality of studies performed with the NOS was evaluated by examining three factors: patient selection, comparability of the study groups, and assessment of measured outcomes. In the item assessing whether follow-up was long enough for outcomes to occur, the cutoff was a priori set at 90 days after major hepatectomy, while regarding the item about follow-up adequacy, a priori rate of 90% was also adopted^18^. A score of 0–9 was allocated to each study, studies with 6 or more scores were considered as high quality.

### Statistical analysis

Review Manager 5.4 was used to analyze the data. For dichotomous data, the odds ratio (OR) with 95% CI was calculated. For continuous data, the mean difference (MD) with 95% CI was calculated. We converted median and range to mean and standard deviation using mathematical models by Hozo *et al*.^26^, and estimated the mean and standard deviation from the sample size, median, and interquartile range using mathematical models by Wan *et al*.^27^. Heterogeneity was assessed by the *I* ^2^ statistic, *I* ^2^ values of 25%, 50%, and 75% were considered as low, moderate, and high, respectively. The random effects model was used when *I* ^2^ of 50% or more, and the fixed effects model was used when *I* ^2^ less than 50%. *P* less than 0.05 was statistically significant. Subgroup analysis and estimation of publication bias were also performed. Subgroup analysis was planned for studies of single-centre, multicenter, RMH cases of 50 or less, RMH cases of more than 50, baseline matching incomplete, and baseline matching complete.

## Results

### Study selection

**Figure 1:**
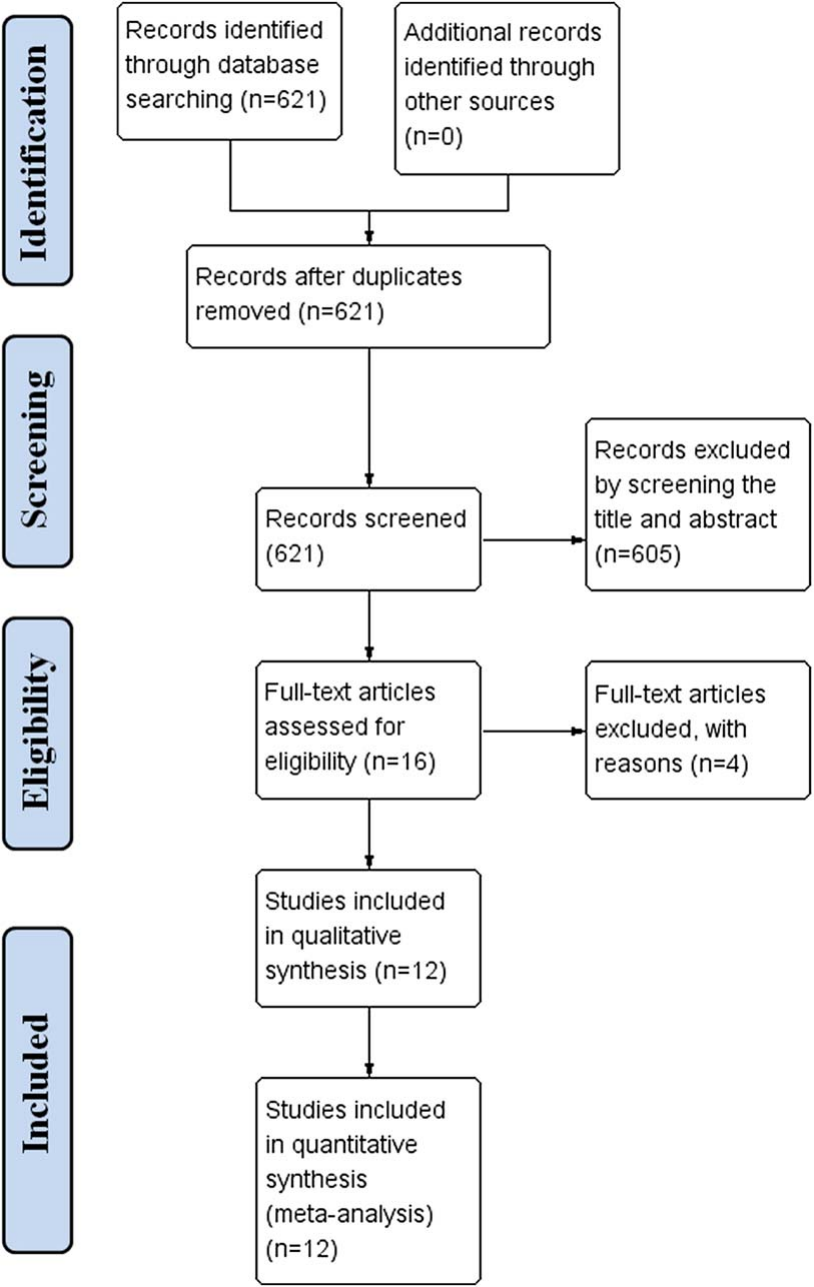
Flow diagram of the study’s selection process.

The flow diagram of study selection procedure is shown in Figure 1. In all, 621 studies were title and abstract-screened for inclusion. Of these, 605 were excluded due to ineligibility or duplicates. Sixteen studies were full text screened. After assessment according to selection criteria and exclusion criteria, we excluded four studies with reasons^28–31^. Finally, we identified 12 studies included in quantitative synthesis^32–43^.

### Quality assessment

All of the included studies were retrospective cohort studies, and thus their quality was assessed using the NOS. All the included studies were of high quality with more than 6 scores, detailed NOS quality assessment for each of the eligible study was shown in Supplementary Table 1, Supplemental Digital Content 4, http://links.lww.com/JS9/B4.

### Study characteristics

The major characteristics of included studies were summarized in Table 1. All studies were published between 2014 and 2022 in conditions of single-centre or multicenter series. The study periods of included studies mostly were after 2010 (range 2005–2022). There were 5 multicenter studies^33,34,40,41,43^, and four of which were international multicenter collaborative studies designed using propensity score matching analysis^33,34,41,43^. Except the four international multicenter studies, the remaining eight included studies were conducted in China (4), USA (2), and Italy (2), respectively. The indications for surgery included both malignant and benign liver disease in all studies except for the study by Hu *et al*.^36^ on hepatectomy for giant haemangioma and the study by Liu *et al*.^37^ on hepatectomy for hepatocellular carcinoma. Four studies^32,35,39,42^ were not adequately matched in reviewed baseline characteristics such as age, BMI, and malignancy rate. Patients in the RMH group were older than those in the LMH group in studies by Fruscione *et al*.^35^ and Wang *et al*.^42^, while RMH group had higher BMI than LMH group in the study by Cai *et al*.^32^. In the study by Mejia *et al*.^39^, cases with malignant tumours were noted more in RMH group than LMH group.

**Table 1:** Study characteristics.

Study	Country	Study period	Study design	Included diseases	Setting/surgeons	Extent of resection	Matched factors	Not matched
Cai *et al*.^32^	China	2015–2020	Single-centre, retrospective	Benign and malignant	3 trained surgeons	LH	1, 2, 4, 5, 6, 7, 8	3
Chiow *et al*.^33^	Singapore	2010–2019	Multicenter, retrospective, PSM	Benign and malignant	21 international centres	RPS	1, 2, 4, 5, 6, 7, 8	—
Chong *et al*.^34^	Singapore	2008–2020	Multicenter, retrospective, PSM	Benign and malignant	29 international centres	RH/ERH	1, 2, 4, 5, 6, 7, 8	—
Fruscione *et al*.^35^	USA	2011–2016	Single-centre, retrospective	Benign and malignant	four trained surgeons	RH/LH/Partial	2, 3, 4, 6, 7	1
Hu *et al*.^36^	China	2011–2017	Single-centre, retrospective	Haemangiomas	a single team of experienced surgeons	RH/LH	1, 2, 6, 7, 8	—
Liu *et al*.^37^	China	2017–2022	single-centre, retrospective	hepatocellular carcinoma (HCC)	surgeons undergoing the same training programs	RH/LH/ERH/ELH/CH	1, 2, 3, 5, 6, 7, 8	—
Marino *et al*.^38^	Italy	2016–2017	single-centre, retrospective	Benign and malignant	a young surgeon	RH	1, 2, 3, 4, 5, 7, 8	—
Mejia *et al*.^39^	USA	2005–2018 (RMH: 2013–2018)	single-centre, retrospective	Benign and malignant	robotic performed by one surgeon, laparoscopic performed by other two surgeons	—	1, 2, 3, 6, 8	7
Spampinato *et al*.^40^	Italy	2009–2012	Multicenter, retrospective	Benign and malignant	4 centres	—	1, 2, 3, 4, 5, 7	—
Sucandy *et al*.^41^	Singapore	2008–2020	Multicenter, retrospective, PSM	Benign and malignant	25 international centres	LH/ELH	1, 2, 4, 5, 6, 7, 8	—
Wang *et al*.^42^	China	2011–2017	single-centre, retrospective	Benign and malignant	a single team of experienced surgeons	RH/LH	2, 3, 4, 5, 6, 7, 8	1
Yang *et al*.^43^	Singapore	2010–2020	Multicenter, retrospective, PSM	Benign and malignant	26 international centres	RAS/CH	1, 2, 4, 5, 6, 7, 8	—

Matching factors: 1, Age; 2, Sex; 3, BMI; 4, ASA score; 5, Previous abdominal surgery; 6, Cirrhosis; 7, Malignancy rate; 8, Tumour size.

ASA, American Society of Anesthesiologists; CH, central hepatectomy; ELH, extended left hepatectomy; ERH, extended-right hepatectomy; LH, left hepatectomy; PSM, propensity score matching; RAS, right anterior sectionectomy; RH, right hepatectomy; RPS, right posterior sectionectomy.

The baseline characteristics of patients in each treatment group were summarized in Table 2. A total of 1657 patients were included in the analysis with 796 (48.0%) undergone RMH and 861 (52.0%) undergone LMH, and 655 (39.5%) were women. However, 7 studies had RMH cases no more than 50. The mean/median age and BMI in each treatment group ranged from 46.5 to 63.0 years and 22.2 to 29.5 kg/m^2^, respectively. The four studies by Chiow *et al*.^33^, Chong *et al*.^34^, Sucandy *et al*.^41^, and Yang *et al*.^43^ included patients with previous hepatic surgery, and the rates of previous liver surgery history in each treatment group ranged from 2.3 to 7.5%. The other four studies by Cai *et al*.^32^, Marino *et al*.^38^, Spampinato *et al*.^40^, and Wang *et al*.^42^ included patients with previous abdominal surgery, but did not reported details specific for history of liver surgery. The study by Liu *et al*.^37^ reported a history of upper abdominal surgery might contraindicate for minimally invasive major hepatectomy. The rates of previous abdominal surgery history reported in each treatment group were between 10.4 and 65.0%. The common comorbidity of cirrhosis in each treatment group ranged from 5.3 to 69.0% in groups included cirrhosis patients. The malignancy rate in each treatment group ranged from 30.8 to 100.0% in studies not specific for malignant diseases. The mean/median size of tumour ranged from 3.5 to 7.1 cm.

**Table 2:** Demographic and baseline characteristics of patients in included studies.

Study	Procedure	Cases	Age (years)	No. (M/F)	BMI (kg/m^2^)	ASA I–II/III–IV	Previous abdominal surgery, *n* (%)	Cirrhosis, n (%)	Malignancy, *n* (%)	Tumour Size (cm)	RH/LH
Cai *et al*.^32^	RMH	25	56.4±9.1	12/13	23.9±3.1	2.1±0.6^a^	6 (24.0)	7 (28.0)	12 (48.0)	5.5±2.3	0/25
	LMH	27	52.7±11.6	18/9	22.2±2.2	1.9±0.6^a^	6 (22.2)	8 (29.6)	15 (55.6)	4.3±1.9	0/27
Chiow *et al*.^33^	RMH	88	60 (51–69)^b^	59/29	—	52/36	27 (30.7)	29 (33.0)	81 (92.0)	3.5 (3.0–5.0)^b^	—
	LMH	88	61 (54–69)^b^	64/24	—	56/31	29 (33.0)	32 (36.4)	83 (94.3)	4.0 (3.0–5.2)^b^	—
Chong *et al*.^34^	RMH	220	61 (52–69)^b^	139/81	—	133/87	75 (34.1)	56 (25.5)	190 (86.4)	5.0 (3.0–7.0)^b^	211/0
	LMH	220	63 (55–71)^b^	144/76	—	128/92	72 (32.7)	50 (22.7)	194 (88.2)	5.0 (3.0–7.5)^b^	212/0
Fruscione *et al*.^35^	RMH	57	58.1±15.7	20/37	28.1±6.3	16/36	—	3 (5.3)	37 (64.9)	—	20/20
	LMH	116	53.2±15.4	52/64	29.5±7.3	30/72	—	7 (6.0)	54 (46.6)	—	46/22
Hu *et al*.^36^	RMH	19	49.2±10.6	2/17	—	—	—	0	0	553.2±122.3^c^	15/4
	LMH	13	46.5±8.9	1/12	—	—	—	0	0	556.2±179.8^c^	8/5
Liu *et al*.^37^	RMH	44	49.82±9.06	29/15	23.29±3.12	—	—	27 (61.4)	44 (100)	—	11/25
	LMH	87	51.49±13.27	61/26	22.28±3.61	—	—	60 (69.0)	87 (100)	—	40/28
Marino *et al*.^38^	RMH	14	58.3±10.4	8/6	28.22±4.59	7/7	8 (57.14)	—	12 (85.7)	4.5±0.5	14/0
	LMH	20	62.3±9.91	11/9	27.9±6.74	12/8	13 (65)	—	20 (100)	4.5±0.8	20/0
Mejia *et al*.^39^	RMH	8	62 (69.5–42.5)^b^	4/4	28.6 (29.9–24.4)^b^	—	—	1 (12.5)	7 (87.5)	6.5 (9.5–4.6)^b^	—
	LMH	13	47 (64–31)^b^	6/7	29.1 (29.6–27.7)^b^	—	—	0	4 (30.8)	5 (8.7–4)^b^	—
Spampinato *et al*.^40^	RMH	25	63 (32–80)^d^	13/12	24 (16.4-21.8)^d^	22/3	16 (64)	—	17 (68)	—	16/7
	LMH	25	62 (33–80)^d^	10/15	25 (20-28.5)^d^	25/0	13 (52)	—	23 (92)	—	15/8
Sucandy *et al*.^41^	RMH	164	62^e^ Median	100/64	—	104/60	41 (25)	26 (15.9)	131 (79.9)	4.65^e^	0/150
	LMH	164	63^e^	105/59	—	101/63	38 (23.2)	33 (20.1)	136 (82.9)	4.1^e^	0/148
Wang *et al*.^42^	RMH	92	54.1±11.2	55/37	24.2±3.9	88/4	10 (10.9)	20 (21.7)	61 (66.3)	7.1±3.3	44/48
	LMH	48	49.4±13.0	24/24	23.7±2.7	47/1	5 (10.4)	9 (18.8)	24 (50.0)	7.0±3.3	19/29
Yang *et al*.^43^	RMH	40	62 (55–68)^b^	32/8	—	29/11	11 (27.5)	18 (45.0)	38 (95.0)	3.8 (3.0–4.9)^b^	—
	LMH	40	62 (54–72)^b^	33/7	—	27/13	13 (32.5)	19 (47.5)	38 (95.0)	3.5 (3.0–5.0)^b^	—

a Mean±SD of ASA score.

b Median (IQR).

c Liver hemangioma volume (cm^3^).

d Median (range).

e Median.

ASA, American Society of Anesthesiologists; IQR, interquartile range; LH, left hepatectomy; LMH, laparoscopic major hepatectomy; No. (M/F), number of patients (male/female); RH, right hepatectomy; RMH, robotic major hepatectomy.

The percentage of patients with cirrhosis was 24.7% (187/757) and 26.7% (218/816) in the RMH and LMH group, respectively. The percentage of patients with malignant disease was 79.1% (630/796) and 78.7% (678/861) in the RMH and LMH group, respectively. Data on the proportion of formal hemihepatectomy were available from nine studies including 1380 patients. Right hemihepatectomy accounted for the 50.2% (331/660) versus 50.0% (360/720) of resection in the RMH and LMH group, respectively, while the proportion of left hemihepatectomy was 42.3% (279/660) versus 37.1% (267/720).

### Primary outcomes

**Figure 2:**
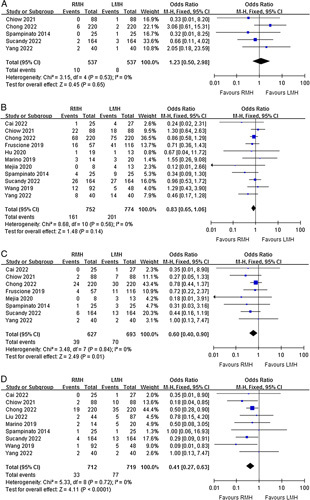
Forest plots of comparison between RMH and LMH on (A) mortality, (B) overall postoperative complications, (C) serious postoperative complications, and (D) conversion to open surgery. LMH, laparoscopic major hepatectomy; RMH, robotic major hepatectomy.

Total 10 studies^32–34,36–38,40–43^ reported the mortality of surgery. Nine studies reported postoperative mortality rates within 90 days and one study^32^ reported perioperative mortality during the same hospitalization. In five^32,36–38,42^ of the ten studies, the mortality rate was 0% for both groups. A meta-analysis of the remaining five studies revealed no significant difference in mortality rates between the two groups (OR=1.23, 95% CI=0.50–2.98, *P*=0.65), and a low heterogeneity was found among the studies (*P*=0.53, I^2^=0%) (Fig. 2A).

Eleven studies reported the incidence of overall postoperative complications. All postoperative complications were defined according to the Clavien–Dindo classification system and recorded for up to 30 days or during the same hospitalization. The overall postoperative complication rate was 21.4% (161/752) in the RMH group and 26.0% (201/774) in the LMH group. There was no significant difference in overall postoperative complications between the RMH and LMH groups (OR=0.83, 95% CI=0.65–1.06, *P*=0.14), and a low heterogeneity was found (*P*=0.56, I^2^=0%) (Fig. 2B).

Eight studies reported the incidence of serious postoperative complications. These serious adverse events were classified according to the Clavien–Dindo classification system of grade 3 or higher. The rate of serious postoperative complications was 6.2% (39/627) in the RMH group and 10.1% (70/693) in the LMH group. The meta-analysis revealed that the incidence of serious postoperative complications in the RMH group was significantly lower than in the LMH group (OR=0.60, 95% CI=0.40–0.90, *P*=0.01), and a low heterogeneity was found (*P*=0.84, I^2^=0%) (Fig. 2C).

Nine studies reported the incidence of conversion to open surgery. The conversion rate was 4.6% (33/712) in the RMH group and 10.7% (77/719) in the LMH group. The meta-analysis revealed that the incidence of conversion to open surgery in the RMH group was significantly lower than in the LMH group (OR=0.41, 95% CI=0.27–0.63, *P*<0.0001), and a low heterogeneity was found (*P*=0.72, I^2^=0%) (Fig. 2D).

### Secondary outcomes

**Figure 3:**
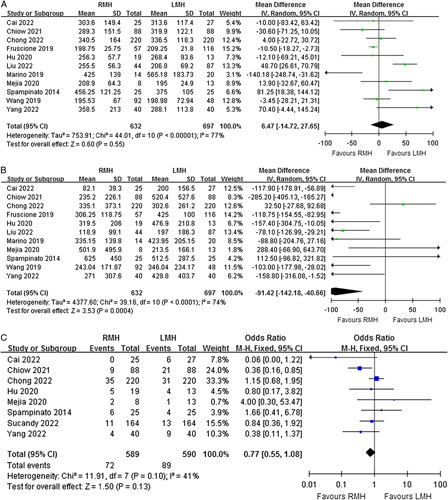
Forest plots of comparison between RMH and LMH on (A) operative time, (B) intraoperative blood loss, and (C) blood transfusion. LMH, laparoscopic major hepatectomy; RMH, robotic major hepatectomy.

Eleven studies reported the operative time. Meta-analysis revealed no statistically significant difference in operative time between the RMH and LMH groups (MD=6.47, 95% CI=−14.72 to 27.65, *P*=0.55) (Fig. 3A). Eleven studies reported the estimated blood loss during surgery. Meta-analysis showed that the intraoperative blood loss of the RMH group was significantly less than that of the LMH group (MD=−91.42, 95% CI=−142.18 to −40.66, *P*=0.0004) (Fig. 3B). However, significant heterogeneities were observed for operative time and intraoperative blood loss among the studies (*P*<0.00001, I^2^=77%; *P*<0.0001, I^2^=74%). Eight studies reported the rate of intraoperative blood transfusion. There was no significant difference in intraoperative blood transfusion between the two groups (OR=0.77, 95% CI=0.55–1.08, *P*=0.13), and a moderate heterogeneity was found (*P*=0.10, I^2^=41%) (Fig. 3C).

**Figure 4:**
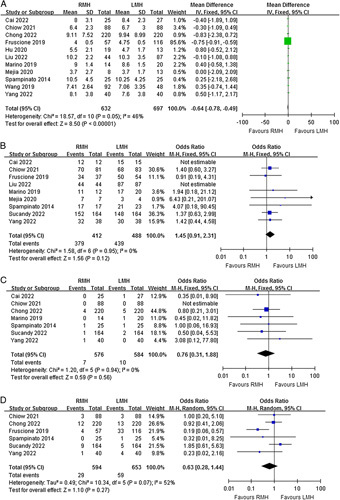
Forest plots of comparison between RMH and LMH on (A) length of hospital stay, (B) R0 resection, (C) reoperation, and (D) readmission. LMH, laparoscopic major hepatectomy; RMH, robotic major hepatectomy.

Eleven studies reported the postoperative length of stay. Meta-analysis revealed that the length of postoperative hospitalization of RMH patients was significantly shorter than that of LMH patients (MD=−0.64, 95% CI=−0.78 to −0.49, *P*<0.00001) (Fig. 4A). The results of the heterogeneity test showed moderate heterogeneity among studies (*P*=0.05, I^2^=46%). Nine studies reported the rate of R0 resection for malignant diseases, seven studies reported the rate of reoperation after primary surgery, and six studies reported the rate of unplanned readmission after primary hospitalization. There were no significant differences between the two surgical approaches in terms of R0 resection (OR=1.45, 95% CI=0.91–2.31, *P*=0.12), reoperation (OR=0.76, 95% CI=0.31–1.88, *P*=0.56), and unplanned readmission (OR=0.63, 95% CI=0.28–1.44, *P*=0.27) (Fig. 4B-D). The results of the heterogeneity test showed low heterogeneity among studies in terms of R0 resection (*P*=0.95, I^2^=0%) and reoperation (*P*=0.94, I^2^=0%). However, there was a high degree of heterogeneity among the studies in unplanned readmission (*P*=0.07, I^2^=52%).

### Subgroup analysis, sensitivity analysis, and publication bias

The subgroup analysis results are partially summarized in Supplementary Table 2, Supplemental Digital Content 4, http://links.lww.com/JS9/B4. The results of the postoperative mortality remained unchanged and had low heterogeneity among all subgroups evaluated. The overall postoperative complications became significant lower in RMH group when analyzed studies no more than 50 RMH cases (OR=0.44, 95% CI=0.23–0.85, *P*=0.01). Although the results of serious postoperative complications was significantly different between RMH and LMH groups in overall analysis, the subgroup analysis results showed consistent effect only in studies of multicenter, more than 50 RMH cases, and baseline matching complete. The lower conversion rate in RMH group became no significant when analyzed studies of single centre (OR=0.41, 95% CI=0.16–1.02, *P*=0.05), or no more than 50 RMH cases (OR=0.69, 95% CI=0.27–1.76, *P*=0.44).

**Figure 5:**
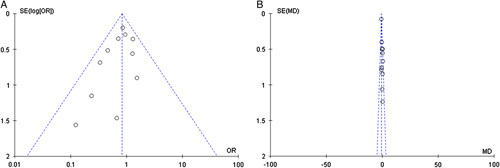
Funnel plots of (A) overall postoperative complications and (B) length of hospital stay for assessing publication bias.

A sequential exclusion of one study at a time was performed for the purpose of sensitivity analysis. The results of this analysis suggested that the pooled value of serious postoperative complications was significantly affected after excluding the study by Sucandy *et al*.^41^ (OR=0.63, 95% CI=0.40–0.99, *P*=0.05), and the heterogeneity among the remaining studies remained low. The pooled value of postoperative length of stay was significantly affected after excluding the study by Fruscione *et al*.^35^ (MD=0.04, 95% CI=−0.34 to 0.43, *P*=0.83), and the heterogeneity among the remaining 10 studies became low (*P*=0.86, I^2^=0%). These sensitivity analysis results indicated that the overall effect size of serious postoperative complications and length of hospital stay was volatile and therefore should be interpreted cautiously. Funnel plot analysis of the overall postoperative complications and hospital stay indicates that the publication bias of these studies was not obvious (Fig. 5).

## Discussion

This meta-analysis compared the perioperative outcomes of RMH to LMH based on available published studies, which included 1657 patients undergone major hepatectomy. To our knowledge, it is the largest meta-analysis to date comparing robotic and laparoscopic liver resections specifically for major hepatectomy. From the perspective of pooled analysis results, this study supports the ongoing trend of adoption of robotic platform to perform complex major hepatectomy. Our findings demonstrate that RMH may lead to lower serious postoperative complications, intraoperative blood loss, and length of hospital stay, while it improves conversion rate with similar operative time. Rates of postoperative mortality, overall postoperative complications, blood transfusion, R0 resection, reoperation, and unplanned readmission are comparable between RMH and LMH. However, the current available published studies comparing RMH with LMH remain limited and all of included literatures are nonrandomized retrospective studies.

As published meta-analyses comparing robotic with laparoscopic liver resections mostly included studies reported both major and minor hepatectomies^12–17^, data from the combined cases of major and minor hepatectomies prevented a valid comparison between RMH and LMH. The meta-analysis by Qiu *et al*.^12^ included nine nonrandomized comparative studies with 254 robotic and 522 laparoscopic hepatectomy cases, which did not show any difference between robotic and laparoscopic liver resection in terms of blood loss, hospital stay, postoperative complications and surgical margin status, except for longer operative time and higher cost in robotic liver resection. The meta-analysis by Kamarajah *et al*.^13^ included 26 nonrandomized comparative studies with 950 robotic and 1680 laparoscopic hepatectomy cases, which did not show any difference in terms of conversion rate, transfusion rate, overall complications, and hospital stay, but less blood loss, longer operative time and lower readmission rate in robotic liver resection. The meta-analysis by Aboudou *et al*.^14^ included 682 patients with robotic liver resection and 1101 patients with laparoscopic liver resection, which did not show any difference in terms of blood loss, hospital stay, and conversion rate, except for longer operative time in robotic liver resection. However, the percentage of major hepatectomy in the robotic group (54.7%) was significantly higher than that in the laparoscopic group (25.2%) in the meta-analysis by Qiu *et al*.^12^. In the meta-analysis by Kamarajah *et al*.^13^, only 20% of included patients had RMH and 14% had LMH in the robotic and laparoscopic group, respectively. In the meta-analysis by Aboudou *et al*.^14^, the subgroup analysis of major and minor hepatectomy on operative time showed no significant difference between the robotic and laparoscopic group. Because a small proportion or different proportion of major hepatectomy between the robotic and laparoscopic group might bias the results in previous meta-analysis, it is not appropriate to directly provide treatment option for major hepatectomy on the basis of the results reported in previous meta-analyses included different difficulty level of hepatectomy.

Previous studies have also attempted to compare robotic and laparoscopic approaches in the setting of major hepatectomy. However, most of which were retrospective case series with small sample. The meta-analysis by Ziogas *et al*.^18^ included seven retrospective cohort studies concerning major hepatectomy (225 RMH and 300 LMH), the pooled results did not show any difference between RMH and LMH in terms of mortality, overall postoperative complications, serious postoperative complications, conversion rate, operative time, blood loss, blood transfusion, length of stay, and R0 resection. As the surgeons’ experience and centres’ volume have an important influence on surgical outcomes thus could bias the comparison of surgeries, the non-significant results pooled from previous studies with small sample did not mean that the RMH had no advantage over LMH, and these results should be interpreted with caution. Another similar meta-analysis by Coletta *et al*.^19^ included eight retrospective cohort studies (244 RMH and 241 LMH), which showed a significantly lower conversion rate and blood loss but longer postoperative hospitalization in RMH as compared to LMH group. However, the aforementioned two analyses included the study by Lee *et al*.^28^ which reported combined outcomes of left hemihepatectomy and left lateral sectionectomy and the study by Tsung *et al*.^29^ which included cases performed by the hand-assist method (placement of hand-port) and hybrid approach. Overall, these previous meta-analyses showed inconstant results and suffered some degree of bias. Moreover, no subgroup analysis was performed in any of these studies due to the small number of patients and events included. Compared to these previous studies, our analyses included more recently published studies from international database and focused specifically on major hepatectomy. Meanwhile, we excluded four articles because the study by Lee *et al*.^28^ reported the combined outcomes of left hemihepatectomy and left lateral sectionectomy, the study by Tsung *et al*.^29^ included cases that laparoscopic resections were performed by the hand-assist method (placement of hand-port) and hybrid approach, the study by Kim *et al*.^30^ compared robotic and laparoscopic living donor right hemihepatectomies, the study by Montalti *et al*.^31^ compared robotic and laparoscopic minor liver resections by parenchymal-preserving method for tumours located in posterosuperior segments. Furthermore, our study performed subgroup analysis in terms of single-centre, multicenter, RMH cases of 50 or less, RMH cases of more than 50, baseline matching incomplete, and baseline matching complete, which might add precision to our comparison of RMH versus LMH.

Our meta-analysis supports the notion that RMH is equally safe as the laparoscopic approach. There was no significant difference regarding the rate of postoperative mortality, overall postoperative complications, reoperation, and unplanned readmission. In addition, in terms of serious postoperative complications, the present meta-analysis showed reduced incidence rate in the RMH group. Despite minimally invasive approach has been widely applied in liver resection, major hepatectomy is technically demanding and hinders the adoption of minimally invasive liver resection in early stage. Heid *et al*. compared the clinical outcomes of LMH and open major liver resection in two Swiss cantonal hospitals, the 90-day mortality rates were reach up to 3% and 7%, respectively^44^. Owing to improvement in equipment and surgical technique over time, the postoperative mortality rates of RMH and LMH in our meta-analysis included the recent data were less than 3% (1.5% and 1.4%, respectively). The postoperative complications are important indicator to surgical quality and prognosis. Consistent with the results of the recent clinical studies^29–33,38–43^, the pooled results of overall postoperative complications for RMH and LMH in our meta-analysis were comparable (21.4% and 26.0%, respectively) and had high homogeneity within included studies. For major hepatectomy, once the occurrence of serious postoperative complications will lead to prolonged hospital stay and even increased risk of reoperation, readmission and mortality^45^. Our meta-analysis showed significant lower serous postoperative complications for RMH compared with LMH in overall analysis. Since massive haemorrhage is one of the most serious complications of minimally invasive major hepatectomy, we speculate the characteristics of robotic platform could contribute to preventing haemorrhage of major vessels. The improved dexterity and three-dimensional view of robotic instruments may not only facilitate control extrahepatic inflow by fine hilar dissection and ligation individual vessels, but also help the safer dissections and control of a series variable hepatic vein in the hepato-caval region. Meanwhile, the rates of reoperation and readmission were with no significant difference, which confirmed the safety and feasibility of RMH.

One interesting finding for our meta-analysis was of decreased conversion rate in the RMH versus LMH. A recent international multicenter study evaluating the risk factors and outcomes of open conversion during minimally invasive major hepatectomies on a total of 3880 patients has observed an overall conversion rate of 4.9% (33/669) in RMH group and 11.4% (366/3211) in LMH group^46^, this finding is near to the value shown for the RMH (4.6%) and LMH (10.7%) in our meta-analysis. Another multicenter study by Cipriani *et al*. comparing robotic and pure laparoscopic hepatectomy based on the difficulty score similarly found that the robotic group exhibited lower conversion rate than the laparoscopic group in the setting of highly difficult surgery^47^. These results suggested that robotic platform may allow an increased proportion of complex major hepatectomy to be finished in a minimally invasive approach^29,48^. Consistently, robotic hepatectomy has been advocated as a technique with the advantage of a shorter learning curve compared to the long learning curve reported for laparoscopic hepatectomy^49,50^. A study reported the number of cases required to surmount the learning curves of laparoscopic and robotic liver resection were 25 and 50, respectively^51^. Although Chong *et al*. showed a significantly lower rate of conversion to open hepatectomy of robotic compared with laparoscopic right and extended-right hepatectomies, a subset analysis further demonstrated that the higher conversion rate of laparoscopic hepatectomy was due to the initial stage of the steeper learning curve of laparoscopic procedure^34^. In other words, there could be no statistical difference in the conversion rate between the robotic and laparoscopic approaches for major hepatectomy when surgeons had surmounted the learning curve of minimally invasive major hepatectomy. Although RMH might be associated with a decreased risk of conversion compared with LMH, converted RMH showed increased postoperative mortality, serious postoperative complications, blood loss, and blood transfusion rate compared with converted LMH^46^. A potential advantage of robotic platform that reducing incidence of conversion to open surgery should be taken into account and prospective studies are warranted to assess whether this effect translates into improved patient outcomes for major hepatectomies in the future.

Major hepatectomy is a more technically demanding and time-consuming procedure, which can attribute to the difficulty in mobilization and exposure of a heavier portion of the liver, and the challenging dissection and transection of liver parenchyma. There was a tendency for patients to experience longer operative time during robotic liver resection because the additional time to dock the robot reported in previous study^37^. However, recent international studies from high volume centres have reported comparable operative time between robotic and laparoscopic surgeries^33,34,41^. Given that the surgeon’s technical proficiency has a major influence on operative time, we speculate the discrepancy of recent results with previous study was caused by the learning curve. Our meta-analysis showed no significant difference in operative time between RMH and LMH, which demonstrated that the difference in operative time in early stage between robotic and laparoscopic liver resection can be overcome along with the maturation of techniques. For malignant liver tumours, resection margin is one of the most important factors indicating the quality of oncologic surgery and an R0 resection is important for reducing recurrence and improving overall survival^52^. In our study, the rates of R0 resection were comparable between RMH and LMH groups, so we opine that oncologic quality was not compromised due to robotic or laparoscopic approaches.

Our meta-analysis showed reduced intraoperative blood loss in RMH, but no significant increase of blood transfusion was found in LMH. The robotic platform has several advantages over the laparoscopic approach such as 7 degrees of freedom and three-dimensional images, which may theoretically facilitate a meticulous haemostasis and avoid injury to major vessels^53^. Although there was a lower blood loss in RMH compared with LMH in our mete-analysis, there was significant heterogeneity among included studies. Unfortunately, the heterogeneity remained moderate to high in our subgroup analyses and we could not further explore the source of heterogeneity as inadequate data. Since the reduced intraoperative blood loss may decrease the incidence of blood transfusion and facilitate the postoperative recovery^54^, whether the reduced blood loss in RMH could translate into a decreased blood transfusion rate and fast postoperative recovery especially for more complex condition such as major hepatectomy need further studies.

Our meta-analysis demonstrated RMH was associated with shorter hospital stay, but there was significant heterogeneity among included studies and the sensitivity analysis results indicated it was volatile. It is well-known that the individual surgeon’s technical proficiency and regional differences of local health care systems have a major influence on postoperative length of hospital stay^34^. Given the heterogeneous proficiency of surgeons and regional differences of included studies, it is difficult to adequately draw conclusion in terms of hospital stay between RMH and LMH based on current studies.

When conducted subgroup analysis of studies no more than 50 RMH cases, the comparable result of overall postoperative complications became favoring RMH and the lower serious postoperative complications in RMH group became no significant. Similarly, the lower conversion rate in RMH group became no significant when analyzed studies of no more than 50 RMH cases. In addition, the lower serious postoperative complications and conversion rate in RMH group became no significant when analyzed studies of single centre. Given the detection power of sample size, so some outcomes studied such as postoperative morbidity and conversion rate may also have been influenced by sample size issues^55^. It suggested that some of the current studies may be underpowered for comparing complex surgical approaches, and further randomized trials with larger numbers of participants are indispensable to clarify surgical outcomes between RMH and LMH with adequate statistical power.

The improved serious postoperative complications were influenced by studies of baseline matching incomplete in subgroup analysis. In addition, our analyses showed serious postoperative complications were lower in RMH group but overall postoperative complications were not reduced in RMH group. Furthermore, the pooled value of serious postoperative complications was significantly affected after excluding the study by Sucandy *et al*.^41^. We speculate the volatile result of postoperative complications may be attributed to a small number of studies focused on major hepatectomy and the inclusion of a relatively small sample. The postoperative morbidities are susceptible to bias, where comparable baseline characteristics and standard definitions are important for accurate comparison of surgical procedures^56^. However, there was short information to perform a more detailed analysis of postoperative complications. Whether RMH could provide benefits in reducing surgical complications, further studies designed to compare RMH and LMH should match complete, unify perioperative management, and use standard outcome definition.

There were several limitations in this study. First, significant heterogeneity was shown in some outcomes, which might be explained by differences in study design, sample size, surgeons’ proficiency, baseline characteristics, healthcare system, postoperative recovery protocol and other factors. Second, variations in sample size among studies were large, and some studies enroled patients during a wide study period, which may have introduced biases due to the advancement in mastering the surgical skills and improvement in surgical instruments. Third, all included studies were carried out in high volume tertiary hospitals, which could be a potential source of bias limiting the generalizability of these findings. Forth, most of the current studies were investigated robotic and laparoscopic approaches in the treatment of both benign and malignant liver neoplasms (hepatocellular carcinoma, colorectal metastasis, cholangiocarcinoma, hemangioma, focal nodular hyperplasia, and so on), there may be specific surgical effects among these cases, we could not obtain enough information to explore these effects and specific surgical indication selection for RMH and LMH should be explored in future studies. All of the factors above might make the comparison results more susceptible to the methodological quality of included studies and lead to high heterogeneity among studies. The cost-effective results and long-term oncologic outcomes are not evaluated in our study as adequate data are missing at present.

In conclusion, the results of our meta-analysis suggest that RMH is associated with comparable short-term surgical outcomes and oncologic adequacy compared to LMH when performed by experienced surgeons at high volume centres. RMH may result in reduced major morbidities, conversion rate, blood loss, and length of hospital stay compared with LMH. In addition, recent studies have addressed the issue of surgical safety of RMH but may not have been sufficiently powered to evaluate the differences in postoperative complications and hospital stay between RMH and LMH. Further randomized studies are required to investigate whether there are advantages of robotic over laparoscopic approach for major hepatectomy.

## Ethical approval

Since the data were from published studies, this study did not need ethical approval.

## Consent

Since the data were from published studies, this study did not need informed written consent.

## Sources of funding

This work was supported by Guangdong Basic and Applied Basic Research Foundation (2021A1515011261).

## Author contribution

Y.Y. contributed to the conception of the study; Y.Y., B.M., S.Z. and D.L. performed the data analyses and wrote the manuscript; J.X. and B.W. helped perform the analysis with constructive discussions.

## Conflicts of interest disclosure

There is no conflict of interest.

## Research registration unique identifying number (UIN)

1. Name of the registry: PROSPERO.
2. Unique Identifying number or registration ID: CRD42023410951.
3. Hyperlink to your specific registration (must be publicly accessible and will be checked): https://www.crd.york.ac.uk/prospero/display_record.php?ID=CRD42023410951.

## Guarantor

Yong Yan.

## Data statement

The data that our review is based on are available in the manuscripts of the included articles.

## Provenance and peer review

Not commissioned, externally peer-reviewed.

## Supplementary Material

SUPPLEMENTARY MATERIAL

## References

[1] Sugawara Y Hibi T. Surgical treatment of hepatocellular carcinoma. Biosci Trends 2021;15:138–141; Epub 2021 Mar 19. 33746184 10.5582/bst.2021.01094

[2] Lafaro KJ Stewart C Fong A. Robotic liver resection. Surg Clin North Am 2020;100:265–281; Epub 2020 Feb 12. 32169180 10.1016/j.suc.2019.11.003

[3] Di Benedetto F Petrowsky H Magistri P. Robotic liver resection: Hurdles and beyond. Int J Surg 2020;82S:155–162; Epub 2020 Jun 3. 32504813 10.1016/j.ijsu.2020.05.070

[4] Viganò L Cimino M Aldrighetti L. Italian Group of Minimally Invasive Liver Surgery (I Go MILS). Multicentre evaluation of case volume in minimally invasive hepatectomy. Br J Surg 2020;107:443–451; Epub 2019 Dec 9. 32167174 10.1002/bjs.11369

[5] Koffron AJ Auffenberg G Kung R. Evaluation of 300 minimally invasive liver resections at a single institution: less is more. Ann Surg 2007;246:385–392; discussion 392-4. 17717442 10.1097/SLA.0b013e318146996c PMC1959347

[6] Strasberg SM. Nomenclature of hepatic anatomy and resections: a review of the Brisbane 2000 system. J Hepatobiliary Pancreat Surg 2005;12:351–355. 16258801 10.1007/s00534-005-0999-7

[7] Ozair A Collings A Adams AM. Minimally invasive versus open hepatectomy for the resection of colorectal liver metastases: a systematic review and meta-analysis. Surg Endosc 2022;36:7915–7937; Epub 2022 Sep 22. 36138246 10.1007/s00464-022-09612-0

[8] Chin KM Linn YL Cheong CK. Minimally invasive vs open major hepatectomies for liver malignancies: a propensity score-matched analysis. J Gastrointest Surg 2022;26:1041–1053; Epub 2022 Jan 21. 35059983 10.1007/s11605-021-05226-4

[9] Swaid F Geller DA. Minimally invasive primary liver cancer surgery. Surg Oncol Clin N Am 2019;28:215–227; Epub 2019 Feb 2. 30851824 10.1016/j.soc.2018.11.002

[10] Wei Chieh AK Chan A Rotellar F. Laparoscopic major liver resections: Current standards. Int J Surg 2020;82S:169–177; Epub 2020 Jul 8. 32652295 10.1016/j.ijsu.2020.06.051

[11] Hamad A Eskander MF Tsung A. What is the value of the robotic platform for major hepatectomies? JAMA Surg 2022;157:445. 35262659 10.1001/jamasurg.2022.0169

[12] Qiu J Chen S Chengyou D. A systematic review of robotic-assisted liver resection and meta-analysis of robotic versus laparoscopic hepatectomy for hepatic neoplasms. Surg Endosc 2016;30:862–875; Epub 2015 Jun 20. 26092026 10.1007/s00464-015-4306-7

[13] Kamarajah SK Bundred J Manas D. Robotic versus conventional laparoscopic liver resections: a systematic review and meta-analysis. Scand J Surg 2021;110:290–300; Epub 2020 Aug 7. 32762406 10.1177/1457496920925637

[14] Aboudou T Li M Zhang Z. Laparoscopic versus robotic hepatectomy: a systematic review and meta-analysis. J Clin Med 2022;11:5831. 36233697 10.3390/jcm11195831 PMC9571364

[15] Hu Y Guo K Xu J. Robotic versus laparoscopic hepatectomy for malignancy: a systematic review and meta-analysis. Asian J Surg 2021;44:615–628; Epub 2021 Jan 16. 33468382 10.1016/j.asjsur.2020.12.016

[16] Gavriilidis P Roberts KJ Aldrighetti L. A comparison between robotic, laparoscopic and open hepatectomy: a systematic review and network meta-analysis. Eur J Surg Oncol 2020;46:1214–1224; Epub 2020 Apr 12. 32312592 10.1016/j.ejso.2020.03.227

[17] Montalti R Berardi G Patriti A. Outcomes of robotic vs laparoscopic hepatectomy: a systematic review and meta-analysis. World J Gastroenterol 2015;21:8441–8451. 26217097 10.3748/wjg.v21.i27.8441 PMC4507115

[18] Ziogas IA Giannis D Esagian SM. Laparoscopic versus robotic major hepatectomy: a systematic review and meta-analysis. Surg Endosc 2021;35:524–535; Epub 2020 Sep 28. 32989544 10.1007/s00464-020-08008-2

[19] Coletta D Levi Sandri GB Giuliani G. Robot-assisted versus conventional laparoscopic major hepatectomies: Systematic review with meta-analysis. Int J Med Robot 2021;17:e2218; Epub 2021 Jan 8. 34196090 10.1002/rcs.2218

[20] Page MJ McKenzie JE Bossuyt PM. The PRISMA 2020 statement: An updated guideline for reporting systematic reviews. Int J Surg 2021;88:105906; Epub 2021 Mar 29. 33789826 10.1016/j.ijsu.2021.105906

[21] Shea BJ Reeves BC Wells G. AMSTAR 2: a critical appraisal tool for systematic reviews that include randomised or non-randomised studies of healthcare interventions, or both. BMJ 2017;358:j4008. 28935701 10.1136/bmj.j4008 PMC5833365

[22] Goh BKP Syn N Koh YX. Comparison between short and long-term outcomes after minimally invasive versus open primary liver resections for hepatocellular carcinoma: a 1:1 matched analysis. J Surg Oncol 2021;124:560–571; Epub 2021 Jun 1. 34061361 10.1002/jso.26556

[23] Goh BKP Lee SY Koh YX. Minimally invasive major hepatectomies: a Southeast Asian single institution contemporary experience with its first 120 consecutive cases. ANZ J Surg 2020;90:553–557; Epub 2019 Nov 12. 31721400 10.1111/ans.15563

[24] Luchini C Veronese N Nottegar A. Assessing the quality of studies in meta-research: review/guidelines on the most important quality assessment tools. Pharm Stat 2021;20:185–195; Epub 2020 Sep 15. 32935459 10.1002/pst.2068

[25] Zhang Y Huang L Wang D. The ROBINS-I and the NOS had similar reliability but differed in applicability: a random sampling observational studies of systematic reviews/meta-analysis. J Evid Based Med 2021;14:112–122; Epub 2021 May 18. 34002466 10.1111/jebm.12427

[26] Hozo SP Djulbegovic B Hozo I. Estimating the mean and variance from the median, range, and the size of a sample. BMC Med Res Methodol 2005;5:13. 15840177 10.1186/1471-2288-5-13 PMC1097734

[27] Wan X Wang W Liu J. Estimating the sample mean and standard deviation from the sample size, median, range and/or interquartile range. BMC Med Res Methodol 2014;14:135. 25524443 10.1186/1471-2288-14-135 PMC4383202

[28] Lee SJ Lee JH Lee YJ. The feasibility of robotic left-side hepatectomy with comparison of laparoscopic and open approach: consecutive series of single surgeon. Int J Med Robot 2019;15:e1982; Epub 2019 Jan 31. 30636179 10.1002/rcs.1982

[29] Tsung A Geller DA Sukato DC. Robotic versus laparoscopic hepatectomy: a matched comparison. Ann Surg 2014;259:549–55. 24045442 10.1097/SLA.0000000000000250

[30] Kim NR Han DH Choi GH. Comparison of surgical outcomes and learning curve for robotic versus laparoscopic living donor hepatectomy: a retrospective cohort study. Int J Surg 2022;108:107000; Epub 2022 Nov 12. 36379423 10.1016/j.ijsu.2022.107000

[31] Montalti R Scuderi V Patriti A. Robotic versus laparoscopic resections of posterosuperior segments of the liver: a propensity score-matched comparison. Surg Endosc 2016;30:1004–13; Epub 2015 Jun 27. 26123328 10.1007/s00464-015-4284-9

[32] Cai JP Chen W Chen LH. Comparison between robotic-assisted and laparoscopic left hemi-hepatectomy. Asian J Surg 2022;45:265–268; Epub 2021 Jun 11. 34120821 10.1016/j.asjsur.2021.05.017

[33] Chiow AKH Fuks D Choi GH International Robotic and Laparoscopic Liver Resection Study Group collaborators. International multicentre propensity score-matched analysis comparing robotic versus laparoscopic right posterior sectionectomy. Br J Surg 2021;108:1513–1520. 34750608 10.1093/bjs/znab321 PMC8743054

[34] Chong CC Fuks D Lee KF. International Robotic and Laparoscopic Liver Resection study group investigators. Propensity Score-Matched Analysis Comparing Robotic and Laparoscopic Right and Extended Right Hepatectomy. JAMA Surg 2022;157:436–444. 35262660 10.1001/jamasurg.2022.0161 PMC8908223

[35] Fruscione M Pickens R Baker EH. Robotic-assisted versus laparoscopic major liver resection: analysis of outcomes from a single center. HPB (Oxford) 2019;21:906–911; Epub 2019 Jan 5. 30617001 10.1016/j.hpb.2018.11.011

[36] Hu M Chen K Zhang X. Robotic, laparoscopic or open hemihepatectomy for giant liver haemangiomas over 10 cm in diameter. BMC Surg 2020;20:93. 32375738 10.1186/s12893-020-00760-5 PMC7204244

[37] Liu L Wang Y Wu T. Robotic versus laparoscopic major hepatectomy for hepatocellular carcinoma: short-term outcomes from a single institution. BMC Surg 2022;22:432. 36528768 10.1186/s12893-022-01882-8 PMC9759871

[38] Marino MV Shabat G Guarrasi D. Comparative study of the initial experience in performing robotic and laparoscopic right hepatectomy with technical description of the robotic technique. Dig Surg 2019;36:241–250; Epub 2018 Mar 14. 29539603 10.1159/000487686

[39] Mejia A Cheng SS Vivian E. Minimally invasive liver resection in the era of robotics: analysis of 214 cases. Surg Endosc 2020;34:339–348; Epub 2019 Apr 1. 30937618 10.1007/s00464-019-06773-3

[40] Spampinato MG Coratti A Bianco L. Perioperative outcomes of laparoscopic and robot-assisted major hepatectomies: an Italian multi-institutional comparative study. Surg Endosc 2014;28:2973–9; Epub 2014 May 23. 24853851 10.1007/s00464-014-3560-4

[41] Sucandy I Rayman S Lai EC. International Robotic, Laparoscopic Liver Resection Study Group Investigators. robotic versus laparoscopic left and extended left hepatectomy: an international multicenter study propensity score-matched analysis. Ann Surg Oncol 2022;29:8398–8406; Epub 2022 Aug 23. 35997903 10.1245/s10434-022-12216-6 PMC9649869

[42] Wang ZZ Tang WB Hu MG. Robotic vs laparoscopic hemihepatectomy: a comparative study from a single center. J Surg Oncol 2019;120:646–653; Epub 2019 Jul 16. 31313324 10.1002/jso.25640

[43] Yang HY Choi GH Chin KM the International Robotic and Laparoscopic Liver Resection Study Group Investigators. Robotic and laparoscopic right anterior sectionectomy and central hepatectomy: multicentre propensity score-matched analysis. Br J Surg 2022;109:311–314. 35139157 10.1093/bjs/znab463 PMC8981979

[44] Heid F Toti J C Balzarotti Canger R. Is laparoscopic major hepatectomy feasible and safe in Swiss cantonal hospitals? Swiss Med Wkly 2021;151:w30044. 34964580 10.4414/smw.2021.w30044

[45] Doussot A Lim C Lahat E. Complications after hepatectomy for hepatocellular carcinoma independently shorten survival: A Western, Single-Center Audit. Ann Surg Oncol 2017;24:1569–1578; Epub 2017 Jan 5. 28058552 10.1245/s10434-016-5746-6

[46] Montalti R Giglio MC Wu AGR. Risk Factors and Outcomes of Open Conversion During Minimally Invasive Major Hepatectomies: An International Multicenter Study on 3880 Procedures Comparing the Laparoscopic and Robotic Approaches. Ann Surg Oncol. 2023;30:4783–4796. 37202573 10.1245/s10434-023-13525-0

[47] Cipriani F Fiorentini G Magistri P. Pure laparoscopic versus robotic liver resections: multicentric propensity score-based analysis with stratification according to difficulty scores. J Hepatobiliary Pancreat Sci 2022;29:1108–1123; Epub 2021 Aug 3. 34291591 10.1002/jhbp.1022

[48] Chong CCN Lok HT Fung AKY. Robotic versus laparoscopic hepatectomy: application of the difficulty scoring system. Surg Endosc 2020;34:2000–2006; Epub 2019 Jul 16. 31312961 10.1007/s00464-019-06976-8

[49] Efanov M Alikhanov R Tsvirkun V. Comparative analysis of learning curve in complex robot-assisted and laparoscopic liver resection. HPB (Oxford) 2017;19:818–824; Epub 2017 Jun 7. 28599892 10.1016/j.hpb.2017.05.003

[50] O’Connor VV Vuong B Yang ST. Robotic minor hepatectomy offers a favorable learning curve and may result in superior perioperative outcomes compared with laparoscopic approach. Am Surg 2017;83:1085–1088. 29391100

[51] Chua D Syn N Koh YX. Learning curves in minimally invasive hepatectomy: systematic review and meta-regression analysis. Br J Surg 2021;108:351–358. 33779690 10.1093/bjs/znaa118

[52] Shukla PJ Barreto SG. Surgery for malignant liver tumors. J Cancer Res Ther 2009;5:154–60. 19841555 10.4103/0973-1482.57119

[53] Becker F Morgül H Katou S. Robotic Liver Surgery - Current Standards and Future Perspectives. Z Gastroenterol 2021;59:56–62; English. Epub 2021 Jan 11. 33429451 10.1055/a-1329-3067

[54] Yan Y Ou C Cao S. Laparoscopic vs. open distal gastrectomy for locally advanced gastric cancer: a systematic review and meta-analysis of randomized controlled trials. Front Surg 2023;10:1127854. 36874456 10.3389/fsurg.2023.1127854 PMC9982133

[55] Yan Y Hua Y Chang C. Laparoscopic versus open pancreaticoduodenectomy for pancreatic and periampullary tumor: a meta-analysis of randomized controlled trials and non-randomized comparative studies. Front Oncol 2023;12:1093395. 36761416 10.3389/fonc.2022.1093395 PMC9905842

[56] de la Plaza Llamas R Hidalgo Vega Á Latorre Fragua RA. The cost of postoperative complications and economic validation of the comprehensive complication index: prospective study. Ann Surg 2021;273:112–120. 30985367 10.1097/SLA.0000000000003308

