# Supplementary figures and images for: Comparison of safety and effectiveness between robotic and laparoscopic major hepatectomy: a systematic review and meta-analysis
Source: Int J Surg. 2023 Sep 14;109(12):4333–46. doi: 10.1097/JS9.0000000000000750 (PMC10720848; doi:10.1097/JS9.0000000000000750)

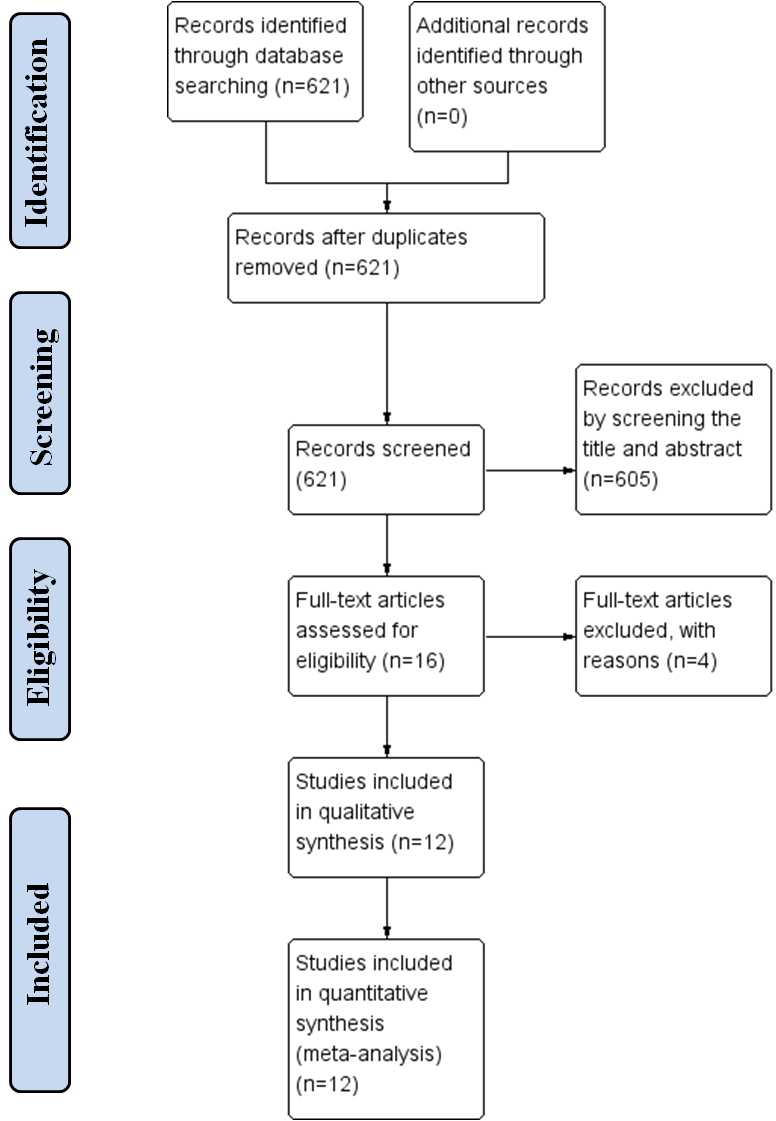


**Fig. 1.** Flow diagram of the study’s selection process.

Supplement: SUPPLEMENTARY MATERIAL [file js9-109-4333-s002.docx]
