# Supplementary material for: Comparison of safety and effectiveness between robotic and laparoscopic major hepatectomy: a systematic review and meta-analysis
Source: Int J Surg. 2023 Sep 14;109(12):4333–46. doi: 10.1097/JS9.0000000000000750 (PMC10720848; doi:10.1097/JS9.0000000000000750)
Supplement: SUPPLEMENTARY MATERIAL [file js9-109-4333-s004.docx]

**Comparison of safety and effectiveness between robotic and laparoscopic major hepatectomy: A systematic review and meta‑analysis**

**Supplementary Table 1** Newcastle-Ottawa scale rating results for included studies

| Study | Representativeness | Selection of non-exposed | Ascertainment of exposure | Outcome not present at start | Comparability on mortality | Comparability on surgeon’s experience | Assessment of outcome | Long enough follow-up (≥90 days) | Adequacy (≥90%) of follow-up | Total score |
| --- | --- | --- | --- | --- | --- | --- | --- | --- | --- | --- |
| Cai 2022 [32] | 1 | 1 | 1 | 1 | 1 | 1 | 1 | 0 | 0 | 7 |
| Chiow 2021 [33] | 1 | 1 | 1 | 1 | 1 | 1 | 1 | 1 | 0 | 8 |
| Chong 2022 [34] | 1 | 1 | 1 | 1 | 1 | 1 | 1 | 1 | 0 | 8 |
| Fruscione 2019 [35] | 1 | 1 | 1 | 1 | 1 | 1 | 1 | 1 | 0 | 8 |
| Hu 2020 [36] | 1 | 1 | 1 | 1 | 1 | 1 | 1 | 1 | 0 | 8 |
| Liu 2022 [37] | 1 | 1 | 1 | 1 | 1 | 1 | 1 | 1 | 0 | 8 |
| Marino 2019 [38] | 1 | 1 | 1 | 1 | 1 | 1 | 1 | 1 | 1 | 9 |
| Mejia 2020 [39] | 1 | 1 | 1 | 1 | 1 | 1 | 1 | 0 | 0 | 7 |
| Spampinato 2014 [40] | 1 | 1 | 1 | 1 | 1 | 1 | 1 | 1 | 0 | 8 |
| Sucandy 2022 [41] | 1 | 1 | 1 | 1 | 1 | 1 | 1 | 1 | 0 | 8 |
| Wang 2019 [42] | 1 | 1 | 1 | 1 | 1 | 1 | 1 | 0 | 1 | 8 |
| Yang 2022 [43] | 1 | 1 | 1 | 1 | 1 | 1 | 1 | 1 | 0 | 8 |

**Supplementary Table 2** Subgroup analysis for single-center/multicenter, RMH less/more than 50 cases, and baseline matching incomplete/complete

| Subgroup/Outcome | No. Studies | No. RMH case | No. LMH case | OR/MD | 95% CI | *P* | *I*^2^ |
| --- | --- | --- | --- | --- | --- | --- | --- |
| Postoperative mortality | | | | | | | |
| Single-center | 1 | 25 | 25 | 0.32 | 0.01, 8.25 | 0.49 | - |
| Multicenter | 4 | 512 | 512 | 1.41 | 0.55, 3.62 | 0.48 | 0% |
| ≤50 RMH cases | 2 | 65 | 65 | 1.00 | 0.17, 5.94 | 1.00 | 0% |
| ＞ 50 RMH cases | 3 | 472 | 472 | 1.31 | 0.47, 3.67 | 0.60 | 14% |
| Baseline matching incomplete | 0 |  |  |  |  |  |  |
| Baseline matching complete | 5 | 537 | 537 | 1.23 | 0.50, 2.98 | 0.65 | 0% |
| Overall postoperative complications | | | | | | | |
| Single-center | 7 | 240 | 262 | 0.67 | 0.42, 1.08 | 0.10 | 0% |
| Multicenter | 4 | 512 | 512 | 0.90 | 0.68, 1.20 | 0.47 | 0% |
| ≤50 RMH cases | 6 | 131 | 138 | 0.44 | 0.23, 0.85 | 0.01 | 0% |
| ＞ 50 RMH cases | 5 | 621 | 636 | 0. 93 | 0.71, 1.21 | 0.59 | 0% |
| Baseline matching incomplete | 4 | 182 | 204 | 0.70 | 0.41, 1.20 | 0.19 | 8% |
| Baseline matching complete | 7 | 570 | 570 | 0.87 | 0.66, 1.14 | 0.32 | 0% |
| Serious postoperative complications | | | | | | | |
| Single-center | 4 | 115 | 181 | 0.49 | 0.19, 1.27 | 0.14 | 0% |
| Multicenter | 4 | 512 | 512 | 0.62 | 0.40, 0.98 | 0.04 | 0% |
| ≤50 RMH cases | 4 | 98 | 105 | 0.42 | 0.13, 1.41 | 0.16 | 0% |
| ＞ 50 RMH cases | 4 | 529 | 588 | 0.62 | 0.40, 0.96 | 0.03 | 0% |
| Baseline matching incomplete | 3 | 90 | 156 | 0.54 | 0.19, 1.52 | 0.24 | 0% |
| Baseline matching complete | 5 | 537 | 537 | 0.61 | 0.39, 0.95 | 0.03 | 0% |
| Conversion to open surgery | | | | | | | |
| Single-center | 5 | 200 | 207 | 0.41 | 0.16, 1.02 | 0.05 | 0% |
| Multicenter | 4 | 512 | 512 | 0.41 | 0.26, 0.67 | 0.0003 | 0% |
| ≤50 RMH cases | 5 | 148 | 199 | 0.69 | 0.27, 1.76 | 0.44 | 0% |
| ＞ 50 RMH cases | 4 | 564 | 520 | 0.36 | 0.22, 0.58 | 0.0001 | 14% |
| Baseline matching incomplete | 2 | 117 | 75 | 0.14 | 0.02, 0.83 | 0.03 | 0% |
| Baseline matching complete | 7 | 595 | 644 | 0.45 | 0.29, 0.69 | 0.0003 | 0% |
| Operative time | | | | | | | |
| Single-center | 8 | 284 | 349 | 6.73 | -20.42, 33.89 | 0.63 | 82% |
| Multicenter | 3 | 348 | 348 | 4.86 | -37.34, 47.07 | 0.82 | 65% |
| ≤50 RMH cases | 7 | 175 | 225 | 18.67 | -18.78, 56.13 | 0.33 | 69% |
| ＞ 50 RMH cases | 4 | 457 | 472 | -9.53 | -16.57, -2.49 | 0.008 | 0% |
| Baseline matching incomplete | 4 | 182 | 204 | -9.29 | -16.57,-2.00 | 0.01 | 0% |
| Baseline matching complete | 7 | 450 | 493 | 11.43 | -25.33, 48.19 | 0.54 | 79% |
| Estimated blood loss | | | | | | | |
| Single-center | 8 | 284 | 349 | -102.61 | -126.21, -79.02 | 0.00001 | 38% |
| Multicenter | 3 | 348 | 348 | -132.03 | -348.47, 84.40 | 0.23 | 92% |
| ≤50 RMH cases | 7 | 175 | 225 | -90.79 | -124.52, -57.05 | 0.00001 | 43% |
| ＞ 50 RMH cases | 4 | 457 | 472 | -109.09 | -208.87, -9.31 | 0.03 | 90% |
| Baseline matching incomplete | 4 | 182 | 204 | -113.68 | -142.14, -85.23 | 0.00001 | 41% |
| Baseline matching complete | 7 | 450 | 493 | -91.47 | -175.48, -7.45 | 0.03 | 79% |
| Blood transfusion | | | | | | | |
| Single-center | 4 | 77 | 78 | 0.80 | 0.35, 1.79 | 0.58 | 44% |
| Multicenter | 4 | 512 | 512 | 0.68 | 0.37, 1.24 | 0.20 | 54% |
| ≤50 RMH cases | 4 | 77 | 78 | 0.80 | 0.35, 1.79 | 0.58 | 44% |
| ＞ 50 RMH cases | 4 | 512 | 512 | 0.68 | 0.37, 1.24 | 0.20 | 54% |
| Baseline matching incomplete | 2 | 33 | 40 | 0.54 | 0.01, 34.32 | 0.77 | 78% |
| Baseline matching complete | 6 | 556 | 550 | 0.81 | 0.57, 1.14 | 0.23 | 34% |
| Length of hospital stay | | | | | | | |
| Single-center | 8 | 284 | 349 | -0.05 | -0.61, 0.50 | 0.85 | 56% |
| Multicenter | 3 | 348 | 348 | -0.27 | -0.92, 0.38 | 0.41 | 0% |
| ≤50 RMH cases | 7 | 175 | 225 | 0.21 | -0.30, 0.72 | 0.42 | 0% |
| ＞ 50 RMH cases | 4 | 457 | 472 | -0.71 | -0.87, -0.56 | 0.00001 | 39% |
| Baseline matching incomplete | 4 | 182 | 204 | -0.72 | -0.88, -0.56 | 0.00001 | 33% |
| Baseline matching complete | 7 | 450 | 493 | 0.03 | -0.41, 0.47 | 0.89 | 0% |
| R0 resection | | | | | | | |
| Single-center | 6 | 129 | 203 | 1.72 | 0.58, 5.16 | 0.33 | 0% |
| Multicenter | 3 | 283 | 285 | 1.39 | 0.83, 2.33 | 0.21 | 0% |
| ≤50 RMH cases | 6 | 130 | 187 | 1.92 | 0.75, 4.89 | 0.17 | 0% |
| ＞ 50 RMH cases | 3 | 282 | 301 | 1.32 | 0.77, 2.26 | 0.32 | 0% |
| Baseline matching incomplete | 3 | 56 | 73 | 1.32 | 0.33, 5.26 | 0.69 | 3% |
| Baseline matching complete | 6 | 356 | 415 | 1.47 | 0.89, 2.41 | 0.13 | 0% |
| Reoperation | | | | | | | |
| Single-center | 3 | 64 | 72 | 0.56 | 0.10, 3.17 | 0.51 | 0% |
| Multicenter | 4 | 512 | 512 | 0.86 | 0.30, 2.49 | 0.79 | 0% |
| ≤50 RMH cases | 4 | 104 | 112 | 0.85 | 0.20, 3.58 | 0.83 | 0% |
| ＞ 50 RMH cases | 3 | 472 | 472 | 0.71 | 0.22, 2.26 | 0.56 | 0% |
| Baseline matching incomplete | 1 | 25 | 27 | 0.35 | 0.01, 8.90 | 0.52 | - |
| Baseline matching complete | 6 | 551 | 557 | 0.83 | 0.32, 2.12 | 0.69 | 0% |
| Readmission | | | | | | | |
| Single-center | 2 | 82 | 141 | 0.20 | 0.07, 0.56 | 0.002 | 0% |
| Multicenter | 4 | 512 | 512 | 1.00 | 0.57, 1.76 | 1.00 | 0% |
| ≤50 RMH cases | 2 | 65 | 65 | 0.26 | 0.04, 1.61 | 0.15 | 0% |
| ＞ 50 RMH cases | 4 | 529 | 588 | 0.74 | 0.28, 1.96 | 0.55 | 67% |
| Baseline matching incomplete | 1 | 57 | 116 | 0.19 | 0.06, 0.57 | 0.003 | - |
| Baseline matching complete | 5 | 537 | 537 | 0.96 | 0.55, 1.68 | 0.89 | 0% |

RMH, robotic major hepatectomy; LMH, laparoscopic major hepatectomy; OR, odds ratio; MD, mean difference; CI, confidence interval.
